# Neurochemical abnormalities in chronic fatigue syndrome: a pilot magnetic resonance spectroscopy study at 7 Tesla

**DOI:** 10.1007/s00213-021-05986-6

**Published:** 2021-10-05

**Authors:** Beata R. Godlewska, Stephen Williams, Uzay E. Emir, Chi Chen, Ann L. Sharpley, Ana Jorge Goncalves, Monique I. Andersson, William Clarke, Brian Angus, Philip J. Cowen

**Affiliations:** 1 Psychopharmacology Research Unit, Department of Psychiatry, University of Oxford, Oxford, UK; 2 Division of Informatics, Imaging and Data Science, Faculty of Medicine, Biology and Health, University of Manchester, Manchester, UK; 3 Wellcome Centre for Integrative Neuroimaging, University of Oxford, Oxford, UK; 4 School of Health Sciences, Purdue University, West Lafayette, IN USA; 5 Wolfson Molecular Imaging Centre, Faculty of Biology, Medicine and Health, University of Manchester, Manchester, UK; 6 Nuffield Department of Medicine, University of Oxford, Oxford, UK; 7 Neurosciences Building, Warneford Hospital, Oxford, OX3 7JX UK

**Keywords:** Chronic fatigue syndrome, MRS, Glutathione, Creatine, Myo-inositol, Anterior cingulate cortex

## Abstract

**Rationale:**

Chronic fatigue syndrome (CFS) is a common and burdensome illness with a poorly understood pathophysiology, though many of the characteristic symptoms are likely to be of brain origin. The use of high-field proton magnetic resonance spectroscopy (MRS) enables the detection of a range of brain neurochemicals relevant to aetiological processes that have been linked to CFS, for example, oxidative stress and mitochondrial dysfunction.

**Methods:**

We studied 22 CFS patients and 13 healthy controls who underwent MRS scanning at 7 T with a voxel placed in the anterior cingulate cortex. Neurometabolite concentrations were calculated using the unsuppressed water signal as a reference.

**Results:**

Compared to controls, CFS patients had lowered levels of glutathione, total creatine and *myo*-inositol in anterior cingulate cortex. However, when using N-acetylaspartate as a reference metabolite, only *myo*-inositol levels continued to be significantly lower in CFS participants.

**Conclusions:**

The changes in glutathione and creatine are consistent with the presence of oxidative and energetic stress in CFS patients and are potentially remediable by nutritional intervention. A reduction in *myo*-inositol would be consistent with glial dysfunction. However, the relationship of the neurochemical abnormalities to the causation of CFS remains to be established, and the current findings require prospective replication in a larger sample.

**Supplementary Information:**

The online version contains supplementary material available at 10.1007/s00213-021-05986-6.

## Introduction

Chronic fatigue syndrome (CFS) is a disabling and persistent condition with prevalence in the UK of up to 2.5% (Prins et al. 2006). Currently, the diagnosis of CFS rests on a specific pattern of clinical symptoms rather than objective physiological markers, which are yet to be determined (Holgate et al. 2011). Better understanding and treatment of CFS can only come from improved knowledge of disease mechanisms, but little is known with certainty about the pathophysiological basis of the disorder. Many symptoms of CFS such as the central pattern of fatigue, cognitive impairment, disturbed sleep, and altered pain sensitivity suggest involvement of the brain (Holgate et al. 2011); accordingly, a number of studies have investigated brain neurochemistry in CFS using magnetic resonance spectroscopy (MRS) (reviewed recently in Van Elzakker et al. (2019) and Shan et al. (2020)).

Early studies using proton MRS at low-field strengths in small groups of CFS participants reported decreased levels of N-acetylaspartate (NAA) (Brooks et al. 2000), a marker of neuronal integrity/function (Rae 2014) and increased levels of choline-containing compounds (Chaudhuri and Behan 2004), suggesting alterations in cell membrane turnover or cellular composition (Rae 2014). More recent MRS work at 3 Tesla (3 T) from one research group demonstrated elevated levels of ventricular lactate in cerebrospinal fluid (CSF) (Mathew et al. 2009; Murrough et al. 2010; Shungu et al. 2012; Natelson et al. 2017), as well as a reduction in the major endogenous free radical scavenger, glutathione (Shungu et al. 2012; Rae and Williams 2017). These findings are consistent with the presence of increased oxidative stress in CFS with a possible secondary component of mitochondrial dysfunction and impaired oxidative energy metabolism (see Maes and Twisk 2010). MRS might also prove a useful tool in demonstrating neuroinflammation in CFS (Van Elzakker et al. 2019). At this point, however, any interpretation of MRS findings is limited, mainly due to the paucity of studies and lack of replication attempts, with many investigations assessing different brain regions (Van Elzakker et al. 2019; Shan et al. 2020).

As far as we are aware, there are no published MRS studies in CFS patients using ultra-high-field (7 T) approaches; however, MRS at 7 T has greater signal to noise ratio (SNR) and increased spectroscopic resolution compared to 3 T, which allows for more precise quantification of a range of neurometabolites (Boer et al. 2011; Godlewska et al. 2017, 2018). Because of the hypothesised abnormalities in oxidative stress and energy metabolism in CFS (see Maes 2009; Morris and Maes 2014), our primary interest was in glutathione and creatine as respective biomarkers of these processes. However, we also examined other metabolites that have been implicated in neuropsychiatric disorders including the amino acid neurotransmitters, glutamate and γ-aminobutyric acid (GABA) as well as the neuronal and glial makers, N-acetylaspartate (NAA) and myo-inositol (Godlewska et al. 2017). We also measured concentrations of glutamine, the metabolite and precursor of glutamate and GABA.

## Methods

### Participants and clinical ratings

Ethical approval for the study was obtained from the National Research Ethics Service Committee (NRES), South-Central Oxford A. Twenty-two patients with CFS (13 females, 9 males, mean age 34.8 years, range 18–60 years) and 13 healthy volunteers (5 females, 8 males, mean age 32.4 years, range 23–54 years) were included in the study after giving full informed written consent and having undergone an MRS scan (three additional CFS patients had a panic attack shortly after entering the scanner, and no MRS data were obtained for these participants). All patients had received a diagnosis of CFS made by an appropriate professional (CFS service or general practitioner) and also met Centers for Disease Control and Prevention (CDC) criteria for CFS (Fukuda et al. 1994), which were administered by a clinically trained member of the research team (BRG). Exclusion criteria for patients included substance use disorder as defined by DSM-5 (determined using the *Structured Clinical Interview for DSM-5*) (First et al. 2015); for healthy volunteers, current or past history of any mental health conditions as defined by DSM-5; and for both groups, contraindications to magnetic resonance (MR) imaging, history of problematic claustrophobia, pregnancy or breast feeding. The current and past mental health history was determined by a structured interview (the *Structured Clinical Interview for DSM-5*, SCID-5).

Mood ratings were measured using the Hamilton Rating Scale for Depression (HAM-D) (Hamilton 1960) and the Beck Depression Inventory II (BDI-II) (Beck et al. 1996), while anxiety ratings were scored using the Spielberger State Anxiety Inventory (STAI) (Spielberger et al. 1993). We also measured fatigue with the Chalder Fatigue Scale (ChFS) (Chalder et al. 1993).

### Magnetic resonance spectroscopy

**Fig. 1:**
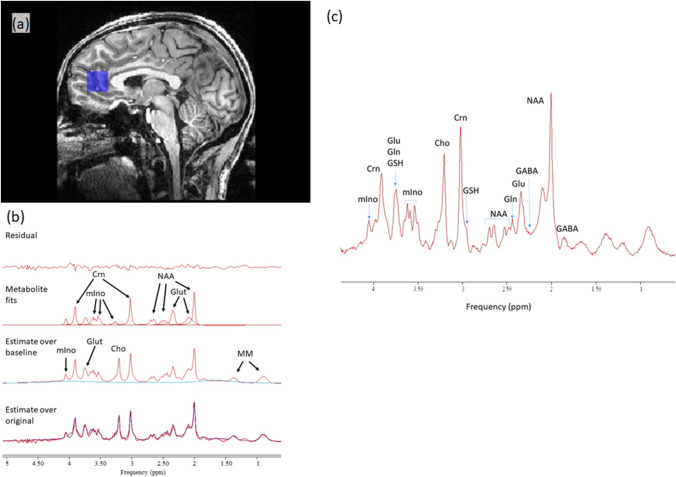
**a** Voxel placement and representative spectrum from the anterior cingulate cortex (ACC). **b** Example spectrum from ACC. The bottom trace shows the raw data, overlaid by a smooth line which is the model fit to 8 metabolite and 2 macromolecule (MM) signals. The middle trace shows the fitted metabolite signals with the major components indicated as NAA, N-acetylaspartate; Glut, glutamate; Crn, creatine + phosphocreatine; Cho, glycerophosphorylcholine, representing choline-containing compounds; and mIno myo-inositol. **c** An example of ACC spectrum. NB. The fit included all components, but only signals which are visually discernible have been labelled, and only a subset of fitted metabolites has been displayed in (**b**)

Participants underwent proton (1H) MRS scanning at the Wellcome Centre for Integrative Neuroimaging in Oxford. Scanning was performed on a 7 T Siemens MAGNETOM scanner (Siemens, Erlangen, Germany) with a Nova Medical 32 channel receive array head coil. Spectra were measured from a voxel in the anterior cingulate cortex (ACC, 20 × 20 × 20 mm) (Fig. 1). The voxel was positioned manually by reference to 1-mm isotropic T1-MPRAGE image. First- and second-order shims were first adjusted by gradient-echo shimming (Shah et al. 2009). The second step involved only fine adjustment of first order shims using FASTMAP (Gruetter and Tkáč 2000). Spectra were acquired using a stimulated echo acquisition mode (STEAM, Gyngell et al. 1991) pulse sequence (TE = 11 ms, TR = 5 s, number of transients = 64) with variable power radiofrequency pulses with optimised relaxation delays (VAPOR, Tkac et al. 1999) water suppression and outer volume saturation (Emir et al. 2012). Unsuppressed water spectra acquired from the same voxel were used to remove residual eddy current effects and to reconstruct the phased array spectra. The choice of acquisition method has minimised any potential T2 relaxation differences between populations (Kuan et al. 2021; Zacharopoulos et al. 2021). Data were exported from the scanner as files and preprocessed using FID-A (Simpson et al. 2017). This included coil combination by amplitude weighting to each channel based on the ratio of maximum signal to the square of the noise (Hall et al. 2014) using the unsuppressed water spectra, removal of motion corrupted averages, frequency and phase drift correction in the time domain and zero-order phase correction. The resulting data were converted into a jMRUI readable format.

Metabolites were quantified in the time domain from the reconstructed spectra using the QUEST routine (Ratiney et al. 2005) in jMRUIv6.0 (www.jmrui.eu Stefan et al. 2009). QUEST fits a linear combination of basis sets to the data, with some additional constraints applied as prior knowledge. Here, basis sets were generated for the STEAM sequence using NMRSCOPE-B (Starcuk and Starcukova 2017) for the following metabolites: creatine (Crn; to represent creatine + phosphocreatine), GABA, glutamate, glutamine, glutathione, glycerophosphorylcholine (to represent total choline-containing compounds), myo-inositol and NAA. Additional signals for macromolecular resonances at 0.9 ppm and 1.3 ppm were also simulated. The linewidths of all the metabolites were constrained to be Lorentzian and equal (on the assumption that the linewidth is largely determined by the field inhomogeneity across the voxel), while the macromolecule signals were fixed to have Gaussian lineshape. For quality assurance, spectra were inspected for obvious artefacts (e.g. poorly suppressed water, lipid contamination), and Crn linewidth was assessed independently of the QUEST fit by using AMARES to fit a single Lorentzian line to the Crn signal at 3.03 ppm. Cramer-Rao lower bounds (CRLB) of the individual metabolite fits were calculated within QUEST. Crn was used as the linewidth reference because it is a well-isolated resonance, rather than NAA for which the C3 glutamate resonance can cause an apparent broadening of the NAA signal if it is not well resolved. The spectra quality requirements were CRLB ≤ 30% and full width half height linewidth less than 2.5 × SD above the group mean.

The MPRAGE images were segmented using SPM to determine cerebrospinal fluid (CSF), grey matter (GM) and white matter (WM) fraction (fGM, fWM, fCSF) in the voxels (Ashburner and Friston 2005). Concentrations were then corrected for CSF with the following formula:

$$\left[\mathrm{Mcorr}\right]\:=\:\left[\mathrm M\right]/(1-\mathrm{fCSF}),$$ 

where [Mcorr] is the corrected concentration, [M] is the water-referenced metabolite concentration from the jMRUI output and fCSF is CSF fraction in the voxel.

Metabolite concentrations were calculated using water as a reference. However, CSF contains very low metabolite concentrations but obviously contributes to the water signal from the region of interest, so it is necessary to correct the metabolite for this apparent dilution effect.

### Statistics

Statistical analyses were performed in SPSS version 22. Differences in metabolite concentrations between patients with CFS and healthy controls in ACC were examined using univariate analysis of variance with gender as a covariate. As this study was exploratory, there was no correction for multiple testing. Baseline differences were tested using independent samples *t*-test for continuous variables and chi-square test for categorical variables. Correlations between individual ratings of fatigue, length of time of illness, and levels of neurochemicals in ACC were carried out using Pearson’s product moment and were not corrected for multiple comparisons.

## Results

The patient and healthy control groups did not differ significantly in terms of age and gender ratio and BMI (see Table 1 for details). The patients had modestly increased scores on measures of depression and anxiety; as expected their ratings of fatigue were substantially higher than controls (Table 1). Given that somatic complaint items in depression scales may cause higher scores in physically unwell individuals (Berry et al. 1984), we included the subscores of the BDI-II (affective, cognitive, and somatic) (Vanheule et al. 2008) in Table 1 to provide a better characterisation of the groups. All participants were non-smokers. The mean age of the patients was 34.8 years, and healthy controls 32.4 years. In 17 patients, a history of viral infection preceded the onset of CFS symptoms. Ten of the CFS patients were taking antidepressant medications, mainly SSRIs (*n* = 6) but also low-dose amitriptyline (*n* = 3) and mirtazapine (*n* = 1) (CFS vs HC: *χ*^*2*^ = 7.159, *p* = 0.007). The list of prescription medications and supplements used by individual patients and controls, as well as their comorbidities, can be found in Supplementary Table 1.

**Table 1:** Demographic data and clinical scores. Values represent numbers or mean (SEM). Abbreviations: *F* females, *M* males, *BMI* body mass index, *HAM-D* Hamilton Rating Scale for Depression, *BDI* Beck Depression Inventory, *STAI-S* Spielberger State Anxiety Inventory–State, *ChFS* Chalder Fatigue Scale

	CFS patients (22)	Healthy controls (13)	Statistics
Current age (years)	34.8 (2.6)	32.4 (3.0)	*t* = 0.581, *p* = 0.565
Gender F/M	13/9	5/8	*χ*^2^ = 0.238, *p* = 0.305
Age at onset (years)	25.2 (9.5)	-	-
Antidepressant medication use	9/22	0/13	
BMI	23.7 (0.8)	24.2 (0.8)	*t* = -0.419, *p* = 0.678
HAM-D	4.5 (1.2)	0.8 (0.4)	*t* = 2.299, *p* = 0.028
BDI total score	7.8 (1.9)	0.5 (0.3)	*t* = 2.923, *p* = 0.006
BDI affective subscore	1.4 (0.4)	0 (0)	*t* = 3.098, *p* = 0.004
BDI cognitive subscore	2.0 (0.6)	0.1 (0.1)	*t* = 2.557, *p* = 0.015
BDI somatic subscore	4.4 (1.2)	0.4 (0.2)	*t* = 2.624, *p* = 0.013
STAI-S	30.4 (2.0)	25.3 (1.4)	*t* = 1.801, *p* = 0.081
ChFS	24.8 (0.7)	10.8 (0.3)	*t* = 12.424, *p* < 0.0001

One spectrum was excluded from the analysis due to failed water suppression leading to a distorted baseline. All other spectra from the 34 subjects were of high quality with consistent quality metrics (% CRLB and linewidth: see Supplementary Table 2), and there were no significant differences in these measures between the two groups. The fitting routine did not always fit all the components in all the spectra: GABA could not be fitted in 1 control spectrum; Gln could not be fitted in 2 controls and 3 CFS spectra; and GSH could not be fitted in 1 CFS spectrum. No values were entered into the statistical analysis for these metabolites in these spectra. An example spectrum from the ACC is shown in Fig. 1. There were no significant between-group differences in GM, WM and CFS content (for values see Supplementary Table 3).

The MRS data revealed several neurochemical abnormalities in ACC in CFS patients, notably in concentrations of creatine, glutathione and myo-inositol, all of which were significantly lower in patients (Table 2). In the CFS patients, there was no significant correlation between score on ratings of fatigue and any of the neurochemicals in ACC. However, there was a moderate significant correlation between NAA and duration of fatigue symptoms (*r* = 0.54, *p* = 0.04). For all correlation values, see Supplementary Table 4.

**Table 2:** Mean (SEM) absolute concentrations (μmol/g) of brain neurochemicals corrected for cerebrospinal fluid (CSF) content in pregenual anterior cingulate cortex (pgACC)

	CFS patients	Healthy controls	*F* value, *p*
Creatine	7.43 (0.24)	8.65 (0.37)	*F* = 8.17, *p* = 0.008
Glutathione	1.17(0.06)	1.48 (0.12)	*F* = 5.94, *p* = 0.021
Glutamate	9.19 (0.21)	9.93 (0.34)	*F* = 4.09, *p* = 0.052
Glutamine	2.09 (0.20)	1.85 (0.21)	*F* = 0.66, *p* = 0.43
GABA	2.33 (0.17)	1.81 (0.12)	*F* = 3.87, *p* = 0.058
NAA	9.64 (0.20)	10.1 (0.36)	*F* = 2.28, *p* = 0.14
Myo-inositol	4.76 (0.18)	5.75 (0.26)	*F* = 8.24, *p* = 0.002

In our study, we used water as reference, similar to other studies (e.g. Murrough et al. 2010; Shungu et al. 2012). Although previous research is not always clear, most studies apparently applied water as reference, apart from Van der Schaaf et al. (2017). As can be seen in Table 2, there was a general tendency for most of the ACC neurometabolites measured to be present in lower concentrations in the CFS patients than controls. This could be explained by a higher tissue water content per unit weight in the brain tissue in patients compared to controls, but cannot be due to changes in tissue composition, since the relative proportions of GM and WM did not differ significantly between the groups and the metabolite concentrations are corrected for the CSF content in the voxel. To explore this further, the analyses were re-run using ratio of each metabolite to NAA concentration, in order to remove water content from the equation (Table 3). NAA was chosen as a reference as there was a significant difference in levels of creatine (the most commonly used reference metabolite) between the CFS patients and controls. With NAA as reference, GABA/NAA ratios were significantly higher in CFS patients relative to controls, while only myo-inositol remained significantly lower.

**Table 3:** Mean (SEM) concentrations (μmol/g) of brain neurochemicals corrected for cerebrospinal fluid (CSF) content in anterior cingulate cortex (ACC) expressed as ratio to NAA

	CFS patients	Healthy controls	*F* value, *p*
Creatine	0.78 (0.03)	0.85 (0.02)	*F* = 3.47, *p* = 0.072
Glutathione	0.12 (0.01)	0.15 (0.01)	*F* = 4.0, *p* = 0.055
Glutamate	0.87 (0.03)	0.89 (0.02)	*F* = 0.81, *p* = 0.78
Glutamine	0.22 (0.02)	0.19 (0.02)	*F* = 0.94, *p* = 0.34
GABA	0.24 (0.02)	0.18 (0.01)	*F* = 5.02, *p* = 0.033
Myo-inositol	0.50 (0.02)	0.57 (0.02)	*F* = 5.62, *p* = 0.024

To account for the use of antidepressants, we added it as a covariate and rerun the analyses. The findings are little changed and are shown in the Supplementary Table 5.

## Discussion

As far as we are aware, this is the first investigation to carry out MRS at 7 T to study brain neurochemistry in patients with CFS. We found several abnormalities in ACC neurochemicals in CFS patients, in particular lowered concentrations of creatine, myo-inositol and glutathione. Interestingly, a decrease in brain glutathione concentration in CFS patients has been reported in a previous MRS investigation by Shungu and colleagues (2012), consistent with the presence of oxidative stress. However, a similar reduction in glutathione may also be present in depressed patients suggesting a lack of diagnostic specificity for this particular abnormality (Shungu et al 2012; Godlewska et al. 2015).

Oxidative stress has long been implicated in the pathophysiology of CFS (Maes and Twisk 2010), and several studies have identified relevant biomarkers in peripheral blood, for example, raised levels of isoprostanes (Richards et al. 2000; Kennedy et al. 2005). Current theories suggest that increased release of cytokines following various stresses, including viral infections, may lead to induction of nitric oxide and the production of excess levels of reactive oxygen and reactive nitrogen species. Lowered levels of glutathione might then represent a consequent depletion of this main cellular anti-oxidant defence thereby allowing further cellular damage by free radicals (Morris et al. 2014).

Chronic oxidative stress can result in mitochondrial dysfunction with resulting deficits in oxidative phosphorylation and energy production (Maes and Twisk 2010). It is therefore of interest that our study revealed a significant decrease in creatine in patients with CFS. In cells, creatine is phosphorylated to form phosphocreatine which acts an energy reserve for adenosine triphosphate (ATP). Creatine deficiency syndromes are associated with low levels of brain creatine and phosphocreatine in MRS studies (Allen 2012). Shungu and colleagues (2012) using phosphorus MRS found no difference in brain levels of high-energy phosphates, including phosphocreatine, in CFS patients compared to controls. As creatine kinase catalyses a reaction close to equilibrium, the only way in which phosphocreatine and ATP can remain unchanged, while total creatine is reduced, would be if there is an increase in free ADP or a decrease in pH, according to the following equation:

$$\begin{array}{c}\mathrm{Cr}+\mathrm{ATP}\leftrightarrow \mathrm{ PCr}+\mathrm{ADP}+{\mathrm{H}}^{+}\\ {K}_{\mathrm{eq}}=\{ [\mathrm{PCr}].[\mathrm{ADP}].[{\mathrm{H}}^{+}]\}/\{[\mathrm{Cr}].[\mathrm{ATP}]\}\end{array}$$

As noted above, it is possible that altered creatine concentration could be related to secondary mitochondrial dysfunction postulated to be involved in the pathophysiology of CFS (Maes and Twisk 2010). The MRS evidence for brain mitochondrial dysfunction CFS is mixed. For example, while Shungu et al (2012) found increased ventricular lactate in CFS, there was no reduction in levels of the key energy molecule, adenosine triphosphate (ATP). However, in addition to its place in energy metabolism, creatine also has an anti-oxidant role (Lawlor et al. 2002; Sestili et al. 2011; Cunha et al. 2014). One possibility, therefore, is that lowered levels of creatine in pgACC in CFS patients might reflect increased consumption of anti-oxidant defences in this particular brain region.

We also found lowered levels of myo-inositol in ACC in CFS patients. Much of the myo-inositol in the brain is present in glia (Leibfritz et al. 1993; Rae 2014) where it may function as an osmolyte (Fisher et al. 2002). Although glial dysfunction, especially involving neuroinflammation and gliosis, is commonly found to be associated with increased myo-inositol levels, there are also reports of decreased myo-inositol concentrations in disorders with hypothesised glial pathology, such as schizophrenia (see Das et al. 2018). Hence, our observation could be consistent with glial dysfunction in this brain region.

Our study suggests that CFS patients may manifest a variety of neurochemical abnormalities in ACC. The location of the voxel in the current study is in the rostral portion of the ACC, also known as the pregenual ACC (pgACC). We chose this voxel because we have carried out previous MRS work in this brain region in patients with depression (Godlewska et al. 2018) whom we thought would form a useful comparison group for patients with CFS, many of whom have depressive symptoms. Also, the pgACC has potential relevance in the pathophysiology of CFS. It is involved in emotional and cognitive processing, and its function is crucial for associative processing requiring integration of various levels of information. It is a critical area for many of the mental functions shown to be disturbed in CFS, such as ability to evaluate and respond to bodily sensations, experience of the body state, pain inhibition, and cognitive processes, such as sustained and selective attention. Also, a recent formulation of the neural circuitry involved in mediating fatigue has implicated the ACC as part of a core system that weighs up the costs and benefits of continued exertion in cognitive and physical tasks (Müller and Apps 2019). Although this theory focuses on the dorsal ACC, a number of studies have shown changes in pgACC function related to the value of effort in decision-making (Polli et al 2005; Ramage et al 2019).

It is important to note that almost half of CFS participants in our study were taking antidepressant medications. Previous studies suggested that antidepressants may influence metabolism and exhibit a glial-inhibiting and anti-inflammatory effect (Sanacora et al. 2002; Zhu et al. 2009; Grace et al. 2014). Although some MRS studies did not observe changes in neurometabolites over the course of SSRI treatment (Godlewska et al. 2015), some other studies did show such an effect (Brennan et al. 2017; Zhang et al. 2015; Taylor et al. 2012); therefore, it is prudent to treat antidepressant use as a potential confounding factor. However, adding antidepressant treatment as a covariate produced little change in our findings (see Supplementary Table 5).

While our findings of several neurochemical abnormalities in ACC in CFS patients are intriguing, they must be received with caution because of the limitations of our study. The use of antidepressants by CFS patients has already been mentioned. The small number of participants, especially in the control group, raises the possibility of false positives type 1 errors, particularly since we made no correction for multiple comparisons. Also, as in many clinical research studies, the healthy control group were in excellent health overall, making it difficult to ascribe neurochemical differences in the patient group specifically to the presence of CFS. It may be relevant, however, that while many of the CFS patients experienced some depressive symptomatology, in a similar 7 T MRS study in patients with major depression, we found no changes in brain neurochemicals in ACC (Godlewska et al. 2018).

In addition, interpretation of neurobiological changes in CFS requires recognising the possibility that abnormalities may be consequences of prolonged inactivity rather than part of causal pathophysiology. However, in the current study, there was no correlation between the abnormal neurochemical findings and current fatigue scores or length of illness (a modest correlation between length of illness and NAA level was positive rather than negative; additionally, this non-a-priori correlation was modest and not corrected for multiple comparisons and therefore should be received with caution. Also, a study of white matter myo-inositol found lower levels in athletes engaged in non-contact sports than in sedentary controls (Schranz et al. 2020).

We also noted that, with the exception of GABA and glutamine, concentrations of all metabolites measured were lower in CFS patients than controls. This gives rise to a concern that our findings could reflect some general difference between the two participant groups, for example, in voxel composition or specific water content. Our data were corrected for CSF content, and we did not see a significant difference in water content between CFS patients and controls. However, as noted above, the proportions of GM and WM in the voxels did not differ between groups, and the data are corrected for CSF content. CSF correction is important as tissue water concentration serves as an internal reference for calculating metabolite concentrations; accounting for individual differences in tissue composition allows for more precise results. Nevertheless, without independent measurements of water content in the brain tissue of patients, we cannot rule out a water dilution effect. However, when measured relative to NAA, there were still decreases in creatine, glutamate, glutathione, and myo-inositol, albeit non-significant for all but myo-inositol. We also compared the absolute water signal from the ACC between the two groups (recognising that instrumental and operational factors can also cause scan to scan differences in this metric) and detected no significant difference.

However, when measured relative to NAA only myo-inositol continued to be lower in CFS patients. Interestingly, when measured relative to NAA, in this analysis, GABA levels were significantly higher in CFS. While GABA is particularly associated with inhibitory interneurons, it is also found in astrocytes and can be present at raised levels in disease states featuring so-called reactive astrocytes that are characterised by elevated glial fibrillary acidic protein (Sonfroniew and Vintners, 2010; Wu et al., 20,104). Therefore, one tentative explanation for our findings is that the neurochemical abnormalities in CFS may be particularly associated with glial cell dysfunction.

It is clearly important that the present pilot findings are replicated prospectively in larger groups of participants. Successful replication could have interesting implications for treatment in that some of the neurochemical abnormalities demonstrated in the present study, that is, in glutathione, creatine and myo-inositol, are potentially treatable by nutritional intervention (Roschel et al. 2021; Schfanfelberg 2021).

## Supplementary Information

Below is the link to the electronic supplementary material.

Supplementary file1 (DOCX 16 KB)

Supplementary file2 (DOCX 13 KB)

Supplementary file3 (DOCX 13 KB)

Supplementary file4 (DOCX 13 KB)

Supplementary file5 (DOCX 13 KB)
