# Supplementary material for: Neurochemical abnormalities in chronic fatigue syndrome: a pilot magnetic resonance spectroscopy study at 7 Tesla
Source: Psychopharmacology (Berl). 2021 Oct 5;239(1):163–71. doi: 10.1007/s00213-021-05986-6 (PMC8770374; doi:10.1007/s00213-021-05986-6)
Supplement: Supplementary file 1 — Supplementary file1 (DOCX 16 KB) [file 213_2021_5986_MOESM1_ESM.docx]

Supplementary Table 1. The list of prescription medications used by individual participants and comorbidities.

|  | Medications/Supplements | Medical conditions (apart from CFS) |
| --- | --- | --- |
|  |  |  |
|  |  |  |
| 1 | Amitriptyline 20mg  Atenolol 10mg (for headaches) | - |
| 2 | Gabapentin 2400mg  Midodrin 15mg | Polycystic ovary syndrome |
| 3 | Gabapentin 2400-3600mg  Mirtazapine 15mg alternate with clonidine 300 mcg every 1-2 days | - |
| 4 | Vit D, multivitamin | - |
| 5 | Citalopram 40mg | - |
| 6 | Vit D, B12, CoQ10, omeprazole | Irritable Bowel Syndrome, hypermobility, bimanual synkinesis |
| 7 | Fish oil, vit D3, vit B, Ca, Mg | - |
| 8 | Multivitamin, vit C, Mg | - |
| 9 | Amitriptyline 25mg (for pain and insomnia)  Doxylamine succinate (for sleep)  Multivitamin | Migraine |
| 10 | Simbacort 2x/day  Montelukast 1x/day  Cerelle (contraceptive) | Asthma |
| 11 | Citalopram 10mg  Trazodone 50mg  Amitriptyline 10mg | - |
| 12 | - | Hypermobility syndrome |
| 13 | Multivitamin, vitD | - |
| 14 | Multivitamin | - |
| 15 | Fluoxetine 40mg  Amitriptyline 20mg  Metformin 2g | Polycystic ovary syndrome |
| 16 | Fluoxetine 40mg  Amitriptyline 10mg  Mebeverine 135mg | Irritable Bowel Syndrome |
| 17 | - |  |
| 18 | Minerals | Graves disease , osteoarthritis (legs) |
| 19 | - | - |
| 20 | Citalopram 30mg | - |
| 21 | Amitriptyline 30mg (for sleep) | Mild asthma |
| 22 | Citalopram 20mg  Omeprazole 20mg |  |
| Healthy Controls |  |  |
| 1 | - | - |
| 2 | - | - |
| 3 | - | - |
| 4 | - | - |
| 5 | Vit C | - |
| 6 | - | - |
| 7 | Vit C, multivitamin, omega-3 fatty acids | - |
| 8 | Levothyroxine 75mcg 5x/week, 50mcg 2x/week | Hypothyroidism (thyroid function within normal range) |
| 9 | - | - |
| 10 | Marvelon | - |
| 11 | - | - |
| 12 | - | Mild asthma |
| 13 | - | - |
