# Supplementary material for: Neurochemical abnormalities in chronic fatigue syndrome: a pilot magnetic resonance spectroscopy study at 7 Tesla
Source: Psychopharmacology (Berl). 2021 Oct 5;239(1):163–71. doi: 10.1007/s00213-021-05986-6 (PMC8770374; doi:10.1007/s00213-021-05986-6)
Supplement: Supplementary file 2 — Supplementary file2 (DOCX 13 KB) [file 213_2021_5986_MOESM2_ESM.docx]

Supplementary Table 2. Quality assurance metrics for MRS. % Cramer-Rao Lower Bounds (CRLB) were calculated as a percentage of the metabolite amplitude for creatine (Crn), GABA, glutamate (Glu), glutamine (Gln), glycerophosphorylcholine (GPC), glutathione (GSH), myo-inositol (mIno) and N-acetylaspartate (NAA). † GABA could not be fitted in 1 control spectrum; *Gln could not be fitted in 2 controls and 3 CFS spectra; ** GSH could not be fitted in 1 CFS spectrum. Full width half height linewidth (LW) is provided for Crn.

| Metab | % CRLB controls (n=13) | | % CRLB CFS (n=21) | |
| --- | --- | --- | --- | --- |
|  | Mean, SD | min-max | Mean, SD | min-max |
| Crn | 0.45±0.10 | 0.32-0.63 | 0.44±0.12 | 0.28-0.66 |
| GABA† | 2.47±0.87 | 1.50-4.48 | 1.80±0.71 | 0.78-3.62 |
| Glu | 0.66±0.14 | 0.44-0.91 | 0.58±0.13 | 0.37-0.90 |
| Gln* | 3.95±1.72 | 1.88-7.21 | 3.58±2.43 | 1.07-10.1 |
| GPC | 0.48±0.09 | 0.92-1.61 | 0.46±0.09 | 0.35-0.79 |
| GSH** | 2.46±1.16 | 1.40-5.88 | 2.54±0.73 | 1.68-4.11 |
| mIno | 0.56±0.11 | 0.42-0.75 | 0.60±0.15 | 0.38-0.90 |
| NAA | 0.37±0.07 | 0.26-0.48 | 0.33±0.06 | 0.26-0.52 |
|  |  |  |  |  |
| Crn LW controls (Hz) | | Crn LW CFS (Hz) | |  |
| Mean, SD | min-max | Mean, SD | min-max |  |
| 9.1±1.4 | 7.6-11.8 | 8.3±1.0 | 6.4-10.0 |  |
