# Supplementary material for: Neurochemical abnormalities in chronic fatigue syndrome: a pilot magnetic resonance spectroscopy study at 7 Tesla
Source: Psychopharmacology (Berl). 2021 Oct 5;239(1):163–71. doi: 10.1007/s00213-021-05986-6 (PMC8770374; doi:10.1007/s00213-021-05986-6)
Supplement: Supplementary file 3 — Supplementary file3 (DOCX 13 KB) [file 213_2021_5986_MOESM3_ESM.docx]

Supplementary Table 3. Mean (SEM) absolute concentrations (μmol/g) of grey matter (GM), white matter (WM) and cerebrospinal fluid (CSF) content in anterior cingulate cortex (ACC).

|  | CFS patients | Healthy controls | t value, p |
| --- | --- | --- | --- |
| GM | 0.788 (0.014) | 0.800 (0.072) | t= -0.507, p= 0.616 |
| WM | 0.101 (0.019) | 0.101 (0.021) | t= 0.107, p= 0.916 |
| CSF | 0.107 (0.013) | 0.097 (0.065) | t= 0.446, p= 0.659 |
