# Supplementary material for: Neurochemical abnormalities in chronic fatigue syndrome: a pilot magnetic resonance spectroscopy study at 7 Tesla
Source: Psychopharmacology (Berl). 2021 Oct 5;239(1):163–71. doi: 10.1007/s00213-021-05986-6 (PMC8770374; doi:10.1007/s00213-021-05986-6)
Supplement: Supplementary file 4 — Supplementary file4 (DOCX 13 KB) [file 213_2021_5986_MOESM4_ESM.docx]

Supplementary Table 4. Correlation between Chalder Fatigue Questionnaire score, length of illness and metabolite levels in the pgACC of patients with CFS.

|  | Chalder Fatigue Questionnaire Score (CFS patients only) | Length of illness (years) |
| --- | --- | --- |
| Creatine | r= -0.26, p=0.254 | r=0.341, p=0.131 |
| Glutathione | r=-0.289, p=0.216 | r=0.168, p=0.479 |
| Glutamate | r=-0.426, p=0.054 | r=0.256, p=0.262 |
| Glutamine | r=-0.014, p=0.959 | r=-0.102, p=0.697 |
| GABA | r=0.102, p=0.661 | r=-0.161, p=0.485 |
| NAA | r=-0.241, p=293 | r=0.451, p=0.04 |
| Myo-inositol | r=-0.279, p=0.221 | r=0.351, p=0.119 |
