# Supplementary material for: Neurochemical abnormalities in chronic fatigue syndrome: a pilot magnetic resonance spectroscopy study at 7 Tesla
Source: Psychopharmacology (Berl). 2021 Oct 5;239(1):163–71. doi: 10.1007/s00213-021-05986-6 (PMC8770374; doi:10.1007/s00213-021-05986-6)
Supplement: Supplementary file 5 — Supplementary file5 (DOCX 13 KB) [file 213_2021_5986_MOESM5_ESM.docx]

Supplementary Table 5. Mean (SEM) absolute concentrations (μmol/g) of brain neurochemicals corrected for cerebrospinal fluid (CSF) content in anterior cingulate cortex (ACC), with gender and antidepressant treatment as covariates.

|  | CFS patients | Healthy controls | F value, p |
| --- | --- | --- | --- |
| Creatine | 7.43 (0.24) | 8.65 (0.37) | F= 7.923, p= 0.009 |
| Glutathione | 1.17(0.06) | 1.48 (0.12) | F= 0.061, p= 0.061 |
| Glutamate | 9.19 (0.21) | 9.93 (0.34) | F= 3.969 p= 0.056 |
| Glutamine | 2.09 (0.20) | 1.85 (0.21) | F= 0.522, p= 0.477 |
| GABA | 2.33 (0.17) | 1.81 (0.12) | F= 4.424, p= 0.044 |
| NAA | 9.64 (0.20) | 10.1 (0.36) | F= 1.596, p= 0.216 |
| Myo-inositol | 4.76 (0.18) | 5.75 (0.26) | F= 10.237, p= 0.003 |
